# Association between household air pollution and nasopharyngeal pneumococcal carriage in Malawian infants (MSCAPE): a nested, prospective, observational study

**DOI:** 10.1016/S2214-109X(21)00405-8

**Published:** 2022-01-18

**Authors:** Mukesh K Dherani, Daniel Pope, Terence Tafatatha, Ellen Heinsbroek, Ryan Chartier, Thandie Mwalukomo, Amelia Crampin, Elena Mitsi, Esther L German, Elissavet Nikolaou, Carla Solórzano, Daniela M Ferreira, Todd D Swarthout, Jason Hinds, Kevin Mortimer, Stephen B Gordon, Neil French, Nigel G Bruce

**Affiliations:** a St Helens and Knowsley Teaching Hospitals NHS Trust, Patterdale Lodge Medical Centre, St Helens, UK; b Department of Public Health, Policy and Systems, University of Liverpool, Liverpool, UK; c Institute of Infection, Veterinary and Ecological Sciences, University of Liverpool, Liverpool, UK; d Malawi Epidemiology and Intervention Research Unit, Lilongwe, Malawi; e Research Triangle Institute International, Durham, NC, USA; f Kamuzu University of Health Sciences, Blantyre, Malawi; g Faculty of Epidemiology and Population Health, London School of Hygiene & Tropical Medicine, London, UK; h Liverpool School of Tropical Medicine, Liverpool, UK; i Malawi-Liverpool-Wellcome Trust Clinical Research Programme, Blantyre, Malawi; j NIHR Mucosal Pathogens Research Unit, Research Department of Infection, Division of Infection and Immunity, University College London, London, UK; k Institute for Infection and Immunity, St George's University of London, London, UK; l BUGS Bioscience, London Bioscience Innovation Centre, London, UK

## Abstract

**Background:**

Household air pollution from solid fuels increases the risk of childhood pneumonia. Nasopharyngeal carriage of *Streptococcus pneumoniae* is a necessary step in the development of pneumococcal pneumonia. We aimed to assess the association between exposure to household air pollution and the prevalence and density of *S pneumoniae* carriage among children.

**Methods:**

The Malawi *Streptococcus pneumoniae* Carriage and Air Pollution Exposure study was a nested, prospective, observational study of children participating in the cluster randomised controlled Cooking and Pneumonia Study (CAPS) in the Karonga Health and Demographic Surveillance System (HDSS) area in northern Malawi. CAPS compared the effects of a cleaner burning biomass-fuelled cookstove (intervention group) with traditional open-fire cooking (control group) on the incidence of pneumonia in children. Eligible children aged 6 weeks or 6 months (those recruited a 6 weeks were also followed up at age 6 months) were identified by the Karonga HDSS centre. Nasopharyngeal swabs were taken to detect *S pneumoniae*, and infant exposure to particulate matter with a diameter of ≤2·5 μm (PM_2·5_) exposure was assessed by use of a MicroPEM device. The primary outcome was the prevalence of nasopharyngeal *S pneumoniae* carriage in all children aged 6 months, assessed in all children with valid data on PM_2·5_. The effects of the intervention stoves (intention-to-treat analysis) and PM_2·5_ (adjusted exposure-response analysis) on the prevalence of *S pneumoniae* carriage were also assessed in the study children.

**Findings:**

Between Nov 15, 2015, and Nov 2, 2017, 485 children were recruited (240 from the intervention group and 245 from the control group). Of all 450 children with available data at age 6 months, 387 (86% [95% CI 82–89]) were positive for *S pneumoniae*. Geometric mean PM_2·5_ exposure was 60·3 μg/m^3^ (95% CI 55·8–65·3) in *S pneumoniae*-positive children and 47·0 μg/m^3^ (38·3–57·7) in *S pneumoniae*-negative children (p=0·044). In the intention-to-treat analysis, a non-significant increase in the risk of *S pneumoniae* carriage was observed in intervention group children compared with control group children (odds ratio 1·36 [95% CI 0·95–1·94]; p=0·093). In the exposure-response analysis, a significant association between PM_2·5_ exposure and *S pneumoniae* carriage was observed; a one unit increase in decile of PM_2·5_ was found to significantly increase the risk of *S pneumoniae* carriage by 10% (1·10 [1·01–1·20]; p=0·035), after adjustment for age, sex, 13-valent pneumococcal conjugate vaccination status, season, current use of antibiotics, and MicroPEM run-time.

**Interpretation:**

Despite the absence of effect from the intervention cookstove, household air pollution exposure was significantly associated with the prevalence of nasopharyngeal *S pneumoniae* carriage. These results provide empirical evidence for the potential mechanistic association between exposure to household air pollution and childhood pneumonia.

**Funding:**

Bill & Melinda Gates Foundation.

## Introduction

Approximately 5·2 million children younger than 5 years died in 2019, most of whom resided in low-income and middle-income countries (LMICs).^1^ Acute lower respiratory infection (ALRI) is a leading cause of mortality in children younger than 5 years in LMICs, accounting for an estimated 14·8% of all deaths in this age group in 2017.^2^ Incomplete combustion of domestic solid fuels, such as wood, dung, and coal, leading to high concentrations of household air pollution is recognised as a major risk factor for childhood ALRI in LMICs.^3^, ^4^, ^5^, ^6^ Over 3 billion people rely on solid fuels for cooking,^7^ a number that has remained constant over the past three decades.^7^ Household air pollution resulting from combustion of these fuels is estimated to cause nearly 1 million deaths from ALRI annually.^8^ *Streptococcus pneumoniae* (pneumococcus) is the most common bacterium implicated in ALRI, and is estimated to cause 30–50% of pneumonia deaths.^9^

**Research in context**

**Evidence before this study**

We consulted two previous systematic reviews conducted by our team on (1) the association between household air pollution and child pneumonia, and (2) the impact of a range of solid fuel and liquid or gaseous fuel interventions on household air pollution and personal exposure. Although household air pollution is an established causal risk factor for child pneumonia, there is scarce direct evidence on the possible mechanisms underlying this association. *Streptococcus pneumoniae* carriage is an important factor in this pathway, and there is indicative evidence of a link between air pollution (both household air pollution and from tobacco smoke) and *S pneumoniae* carriage, although these studies do not include objective personal exposure measurements.

**Added value of this study**

By being nested within a randomised controlled trial of an improved cookstove intervention in Malawi (the CAPS), this study allowed both intention-to-treat and exposure-response analyses of exposure to household air pollution and *S pneumoniae* carriage, with exposure measured using one of the largest datasets of personal infant particulate matter (PM)_2·5_ data reported to date. The intervention stove did not affect exposure to household air pollution or *S pneumoniae* carriage, but we found a positive association between pneumococcal carriage and PM_2·5_ exposure concentrations.

**Implications of all the available evidence**

Our study provides novel objective evidence on the role of household air pollution in facilitating *S pneumoniae* carriage in infants, which is potentially an important mechanism that leads to increased susceptibility to pneumonia.

The pathways by which exposure to household air pollution increases the risk of ALRI, including pneumococcal pneumonia, in children are not well understood. One proposed mechanism is the increased colonisation of the upper airway by pathogens.^10^ Nasopharyngeal carriage of *S pneumoniae* is considered to be an obligate step for pneumococcal disease to develop,^11^ and it is highly correlated with an increased incidence of pneumonia. In sub-Saharan Africa, the region with the highest incidence of pneumonia, nasopharyngeal carriage of *S pneumoniae* in children occurs early and the prevalence often exceeds 85%, which is substantially higher than that observed in the UK and USA, where the prevalence is approximately 50%.^12^ As shown in studies of ambient air pollution^13^ and tobacco smoke,^14^ airborne pollutants are associated with an increased risk of *S pneumoniae.* However, the association between household air pollution from solid fuel use and *S pneumoniae* remains to be elucidated. To our knowledge, only two studies have examined the association between household air pollution and *S pneumoniae* carriage in low-income countries.^15^, ^16^ The results of these studies are inconsistent and difficult to interpret, with limited availability of child exposure measurements. Direct measurement of child exposure to household air pollution (ideally particulate matter with a diameter of ≤2·5 μm [PM_2·5_]) is required to help elucidate causality.

The Cooking and Pneumonia Study (CAPS), a cluster randomised controlled trial in Malawi,^17^ assessed the effectiveness of an improved combustion cookstove intervention in reducing child ALRI through reductions in household air pollution. CAPS provided an opportunity to investigate the association between directly measured exposure to household air pollution in children and nasopharyngeal carriage of *S pneumoniae* through the leveraged, prospective, Malawi *Streptococcus pneumoniae* Carriage and Air Pollution Exposure (MSCAPE) study, which was integrated into the CAPS randomised controlled trial structure.

MSCAPE recruited children included in CAPS to examine the effect of exposure to household air pollution (specifically PM_2·5_, as this is the most important pollutant associated with respiratory disease) on the prevalence and density of nasopharyngeal *S pneumoniae* carriage. The aims of the MSCAPE study were to summarise the effect of the improved combustion stove on PM_2·5_ exposure and *S pneumoniae* carriage in children, and to describe exposure-response associations between PM_2·5_ exposure in children and the prevalence and density of nasopharyngeal *S pneumoniae* carriage.

## Methods

### Study design and participants

MSCAPE was a nested, prospective, observational study done within the Karonga Health and Demographic Surveillance System area (HDSS) in northern Malawi (Karonga), using the CAPS sampling frame. The HDSS routinely conducts surveys of individuals (approximately 40 000) from over 8000 households (clusters of each comprising 20–30 households).^18^ The CAPS randomly assigned 100 HDSS clusters into intervention (an improved, cleaner-burning, biomass-fuelled, combustion cookstove) and control (traditional cooking stove) groups. The randomisation was computer-generated by the trial statistician using dummy codes to represent the intervention and control groups. Randomisation was stratified by study site, distance from health centre, and size of cluster. Eligible households included at least one child younger than 54 months. The intervention was a fan-assisted advanced combustion stove (Philips HD4012LS; Philips South Africa, Johannesburg, South Africa), which is a gasifier stove that burns solid fuels or biomass in a ceramic-lined, stainless-steel combustion chamber. Combustion of these fuels is more efficient than those used in traditional stoves (typically a three-stone open fire, which is the most common method of cooking in rural Malawi), and consequently, should reduce the amount of household air pollution young children are exposed to when their mothers are cooking in the kitchen. Each household in the intervention group was given two improved combustion stoves to accommodate cooking for a typical family. Hence CAPS was designed to test the hypothesis that replacing open fires with these improved cookstoves would reduce pneumonia incidence in young children.^17^ By nesting the current study within the CAPS, we sought to take advantage of the potential for a reduction in household air pollution exposure in the intervention group, free of other confounding factors.

MSCAPE incorporated a prospective design nested within the established infrastructure of the CAPS, which recruited children born into the CAPS households (intervention and control groups) at age 6 weeks (thus MSCAPE children were those from the CAPS). MSCAPE was conducted towards the end of CAPS. Our original cohort was to include children aged 6 weeks, who were to be followed up to the age of 6 months; however, due to CAPS concluding its field data phase earlier than originally expected (and control households being allocated the intervention stoves as per protocol), insufficient numbers of children aged 6 weeks were recruited to achieve the required numbers at follow-up. For this reason, the sample size of children aged 6 months was increased cross-sectionally.

Two field teams, each with two fieldworkers, recruited children aged 6 weeks or 6 months identified for MSCAPE. Eligible children were those aged 6 weeks and 6 months from CAPS homes, details of which are published in full previously.^17^ Eligible children were identified by the Karonga HDSS centre, which generated lists from CAPS (children enrolled at age 6 weeks were also followed up when they reached the age of 6 months).

Written informed consent was obtained after the parents or guardians of children were provided with a full explanation of the MSCAPE study rationale and methods. The study was approved by the Malawi College of Medicine Research and Ethics Committee (P.08/15/1794) and the Central Ethics Committee of the University of Liverpool (RETH000839).

### Procedures

Data were collected in two phases. First, a face-to-face survey was done to confirm household demographic details and to obtain informed consent. Second, after approximately 48 h of the first visit, nasopharyngeal swabs to detect *S pneumoniae* and objective measurements of household air pollution were collected, and information about sociodemographic and household covariates were also collected by questionnaire. Survey data were collected from mothers of the study children by fieldworkers with electronic tablets. The surveys gathered information on household characteristics, household sociodemographics, cooking practices (eg, the type of stove and fuels used), child vaccination status (counterchecked via personal health records), and ongoing use of antibiotics. Data collection was completed twice for children recruited at the age of 6 weeks (at 6 weeks and then again at 6 months) and once for those recruited at the age of 6 months.

To measure the prevalence and density of nasopharyngeal carriage of *S pneumoniae*, nasopharyngeal swabs were taken from the study children according to a standardised paediatric protocol. This protocol involved inserting a sterile paediatric calcium alginate swab into the nostril of each child to the back of nasopharynx, rotating it five times, then placing it immediately into 1 mL of skimmed milk tryptone glucose glycerol (STGG) medium. Samples were then labelled and placed into cold storage for transport to the Karonga HDSS laboratory and stored at –80°C (ie, within 6 h of collection). Pneumococcal detection was ascertained from the nasopharyngeal swab samples in two stages. First, pneumococcal cultures were isolated at the Karonga HDSS laboratory according to standardised methods,^19^ and 6-mm optochin discs (5 μg optochin; Oxoid, Hampshire, England) were used to confirm the presence of *S pneumoniae* via morphology and optochin sensitivity. Second, semi-quantitative PCR targeting the autolysin gene was done using the original inoculated STGG medium of all *S pneumoniae*-positive samples (Liverpool School of Tropical Medicine, Liverpool, UK) to measure density.

Personal exposure to PM_2·5_ in the study children was assessed by use of RTI International MicroPEM technology (RTI International, Research Triangle Park, NC, USA). MicroPEM devices are small, mobile phone-sized instruments that simultaneously measure integrated PM_2·5_ exposure, using an active pump for aerodynamic sizing with filter-based sample collection, and real-time exposure patterns to PM_2·5_ using laser light-scattering nephelometry. During periods of measurement (up to 48 h) the MicroPEM instruments were located in the pockets of specially tailored aprons worn by the child's mother or guardian when carrying the study children. When children were not being carried, including when they were asleep, the instruments were placed within 1 m of the child. After the 48-h recording period, nephelometer data were downloaded onto a study laptop, and the filters were removed and placed in cold storage (–20°C). Filters were then shipped to RTI International for gravimetric analysis, where these data were combined with the nephelometric data. Calibration of instrumentation and data quality control (using field blank filters) were assessed according to standardised RTI protocols.^20^

### Outcomes

The primary outcome was the prevalence of nasopharyngeal *S pneumoniae* carriage in all children aged 6 months. A secondary outcome of *S pneumoniae* density was assessed in all children who were identified as positive for nasopharyngeal carriage of *S pneumoniae*.

### Statistical analysis

The sample size was powered to detect a conservative 10% absolute reduction (from 60% to 50%) in prevalence of *S pneumoniae* at the age of 6 months between the intervention and control groups. A total of 400 children were required in each of the intervention and control groups to achieve 80% power with 5% statistical significance (two-tailed p<0·05), assuming a 15% loss to follow-up or refusal to participate,^21^ with an intraclass correlation coefficient of 0·12 to allow for clustering in the design of CAPS.

An intention-to-treat analysis was done to quantify the effect of the intervention (ie, the advanced combustion cookstove) on the prevalence of nasopharyngeal *S pneumoniae* carriage in children at the age of 6 weeks, 6 months, and in both age groups combined. For comparisons between groups involving PM_2·5_ exposure, a non-parametric test (Mann-Whitney) was used due to skewed data. A multilevel (individual and cluster) mixed-effects logistic regression model was used with (1) intervention versus control status and visit (age 6 weeks and 6 months) as fixed effects, and (2) sample clusters and study child included as random variables. Other covariates included in the model were sex, any dose of 13-valent pneumococcal conjugate vaccine (PCV13), season (wet or dry), ongoing use of antibiotics, and duration of MicroPEM run-time. The Huber–White Sandwich approach was used to calculate robust SEs.^22^ Datapoints with missing information on the primary outcome of nasopharyngeal carriage were excluded from the model. All analyses were done using Stata version 14.1.

To summarise the association between exposure to household air pollution and the prevalence of nasopharyngeal *S pneumoniae* carriage, an exposure-response analysis was done with PM_2·5_ data and the presence or absence of *S pneumoniae*. Measurements with recording periods of less than 24 h were excluded. Household air pollution exposure was defined by decile of PM_2·5_ distribution as a fixed effect. This definition was chosen in preference to the frequency distribution because of the extensive variation in individual amounts of exposure. We did multilevel logistic regression analysis to summarise the effect of a unit change in decile of PM_2·5_ distribution on the risk of incident *S pneumoniae,* using the same covariates as for the intention-to-treat analysis. As there was evidence of an association between duration of exposure and concentration of PM_2·5_, duration of exposure was included in the exposure-response analysis for all participants. To examine the possible effect of differing exposure durations, an exposure-response analysis was also done in participants with a duration of exposure of 44–52 h, with adjustment for the same covariates (other than exposure duration).

To estimate the effect of PM_2·5_ exposure on the density of *S pneumoniae* (expressed as colony-forming units [CFU]/mL) as a continuous dependent variable, the data were log-transformed due to the markedly positively skewed distribution. *S pneumoniae* density data were discarded for positive samples that had a cycle threshold value of 40 or higher. A multilevel generalised linear model was used, with Gaussian family and identity link function to describe linear associations, adjusting for sex, PCV13 vaccination status, season, current antibiotic use, and actual MicroPEM run-time.

### Role of the funding source

The funder of the study had no role in study design, data collection, data analysis, data interpretation, or writing of the report.

## Results

**Figure 1:**
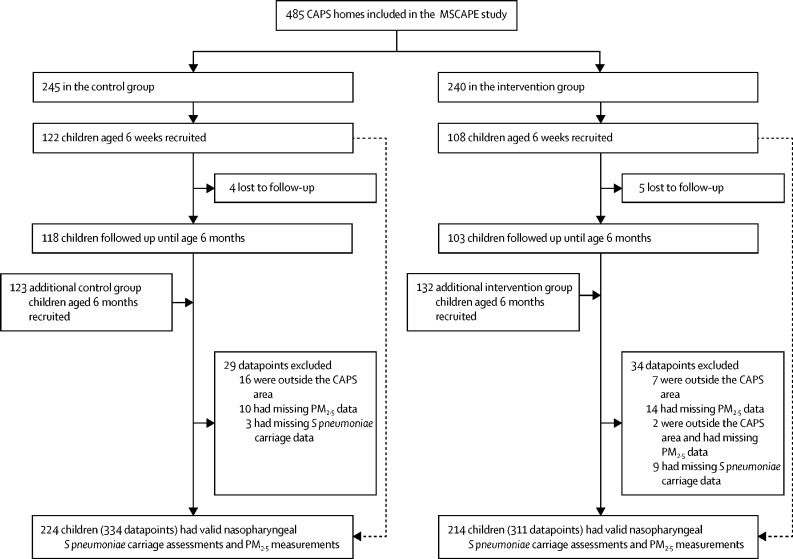
Recruitment of the MSCAPE study children from within the CAPS CAPS=Cooking And Pneumonia Study. MSCAPE=Malawi *Streptococcus pneumoniae* Carriage and Air Pollution Exposure. PM=particulate matter.

Between Nov 15, 2015, and Nov 2, 2017, 485 children were recruited (240 from the intervention group and 245 from the control group), with 230 children recruited at the age of 6 weeks and an additional 255 children recruited at the age of 6 months, generating a total of 706 datapoints (figure 1). Of the 230 children recruited at the age of 6 weeks, almost all (221 [96%]) were followed up at the age of 6 months. Overall, 645 valid outcome and exposure datapoints were available for the final analysis (figure 1). In total, 61 datapoints (29 from the control group and 31 from the intervention group) were excluded: 24 did not have valid exposure data, 12 had missing *S pneumoniae* samples, 23 were outside the CAPS area, and two had both missing exposure data and were outside the CAPS area and were excluded. The median sampling period was 47·8 h (IQR 8·1) in children in the control group and 48·0 (6·8) in those in the intervention group.

Characteristics of the study children at the age of 6 weeks and 6 months (collected during the first study visit), stratified by intervention group, are shown in table 1. Nearly a quarter of children aged 6 weeks (52 [23%] of 230), and almost all children aged 6 months (251 [98%] of 255) had been vaccinated with at least one dose of PCV13, and over two-thirds (350 [74%] of 476) had completed the full course of three doses by 6 months of age. For children aged 6 weeks, there were no notable differences between the intervention and control groups in terms of sex, number of PCV13 vaccine doses received, antibiotic use, and season of assessment. Aside from the higher proportion of boys in the intervention group than in the control group, all other characteristics for children aged 6 months were similar between the two groups.

**Table 1:** Characteristics of included children at baseline

	**Children recruited at age 6 weeks**		**Children recruited at age 6 months**	
	Control group (n=122)	Intervention group (n=108)	Control group (n=123)	Intervention group (n=132)
**Sex**				
Girls	68 (56%)	57 (53%)	70 (57%)	55 (42%)
Boys	54 (44%)	51 (47%)	53 (43%)	77 (58%)
**Number of PCV13 vaccine doses received**				
Any	28 (23%)	24 (22%)	121 (98%)	130 (99%)
None	94 (77%)	84 (78%)	2 (2%)	2 (2%)
1	28 (23%)	24 (22%)	11 (9%)	9 (7%)
2	NA	NA	21 (17%)	23 (17%)
3	NA	NA	89 (72%)	98 (74%)
**Main cookstove used in past 48 h**				
Philips	1 (1%)	48 (44%)	5 (4%)	56 (42%)
Open fire	114 (93%)	59 (55%)	110 (89%)	72 (55%)
Charcoal stove	7 (6%)	1 (1%)	8 (7%)	4 (3%)
**Current antibiotic use**				
Yes	12 (10%)	17 (16%)	24 (20%)	18 (14%)
No	110 (90%)	91 (84%)	99 (81%)	114 (86%)
**Season**				
Wet	114 (93%)	92 (86%)	85 (69%)	90 (68%)
Dry*	8 (7%)	15 (14%)	38 (31%)	42 (32%)

Data are n (%). NA=not applicable. PCV13=13-valent pneumococcal conjugate vaccine.

* Defined as between June and September.

Most control group households (224 [91%] of 245) primarily reported using traditional open fires with wood for cooking, as did more than half of the intervention group households (131 [55%] of 240). A similar proportion of intervention group households with study children aged 6 weeks (59 [55%] of 108) and 6 months (72 [55%] of 132) reported having stopped using the intervention stoves.

Of the 450 children with data on *S pneumoniae* carriage at 6 months of age, 387 (86% [95% CI 82–89]) were positive for nasopharyngeal *S pneumoniae*. The prevalence of *S pneumoniae* carriage in children from intervention households was higher than in those from control group households (adjusted odds ratio [OR] 1·55 [95% CI 0·93–2·59]), although this difference was not significant (p=0·094; table 2).

**Table 2:** Association between the advanced combustion cookstove intervention and prevalence of nasopharyngeal *Streptococcus pneumoniae* carriage in the intention-to-treat population

	**Children aged 6 weeks (n=218)**		Children aged 6 months (n=450)*		All children (n=668)*	
	OR (95% CI)	p value	OR (95% CI)	p value	OR (95% CI)	p value
Crude estimates	1·27 (0·72–2·25)	0·40	1·46 (0·88–2·43)	0·15	1·33 (0·97–1·81)	0·072
Adjusted estimates†	1·23 (0·72–2·10)	0·46	1·55 (0·93–2·59)	0·094	1·36 (0·95–1·94)	0·093

* Analysis accounts for non-independence of repeated measurements for children recruited at the age of both 6 weeks and 6 months.

† Adjusted for age, sex, number of any vaccine doses received, current antibiotic use, and season.

Of the 218 children aged 6 weeks for whom data on *S pneumoniae* carriage were available (2 children had missing data), 87 (40% [95% CI 33–46]) were positive for nasopharyngeal *S pneumoniae*. As with children aged 6 months, the prevalence of *S pneumoniae* carriage was slightly higher in children from intervention group households than in those from control group households (adjusted OR 1·23 [95% CI 0·72–2·10]), although, this difference was not significant (p=0·46; table 2).

In the intention-to-treat analysis of children aged 6 weeks and 6 months combined, we found a 36% increased risk of nasopharyngeal *S pneumoniae* carriage in those from intervention group households compared with those from control group households (OR 1·36 [95% CI 0·95–1·94]), although this difference was not significant (p=0·093; table 2).

To assess the effect of the intervention cookstoves on the concentration of household air pollution, the distributions of PM_2·5_ exposure were compared between children from intervention group and control group households. Individual variability in PM_2·5_ exposure was high in both 6-week and 6-month age groups, with exposure ranging from 3·9 μg/m^3^ to 617·0 μg/m^3^ (median 49·6 μg/m^3^). In accordance with the findings from the intention-to-treat analysis, geometric mean PM_2·5_ exposure was slightly higher in children recruited at the age of 6 weeks and 6 months from intervention group households than in those from control group households, indicating an absence of effect from the improved combustion stove (table 3). Average exposure was greater in children aged 6 months (n=429; 58·3 μg/m^3^ [95% CI 54·1–62.7) than in children aged 6 weeks (n=216; 39·8 μg/m^3^ [36·1–43·8]), irrespective of age at recruitment.

**Table 3:** Personal PM_2·5_ exposure in children by age at recruitment and study group

	**Children recruited at age 6 weeks**				**Exposure in children recruited at age 6 months**		**Exposure in all children**	
	Exposure at recruitment		Exposure at age 6 months		Participants	Geometric mean (95% CI), μg/m^3^	Participants	Geometric mean (95% CI), μg/m^3^
	Participants	Geometric mean (95% CI), μg/m^3^	Participants	Geometric mean (95% CI), μg/m^3^				
Control group	115	38·23 (33·1–44·2)	110	52·5 (45·4–60·9)	109	59·2 (51·4–68·2)	334	49·0 (45·0–53·3)
Intervention group	101	41·59 (36·6–47·3)	97	62·1 (52·7–73·2)	113	60·1 (52·0–69·4)	311	53·9 (49·4–58·7)

This analysis was done in children with valid nasopharyngeal *Streptococcus pneumoniae* carriage assessments and PM_2·5_ measurements. PM=particulate matter.

**Figure 2:**
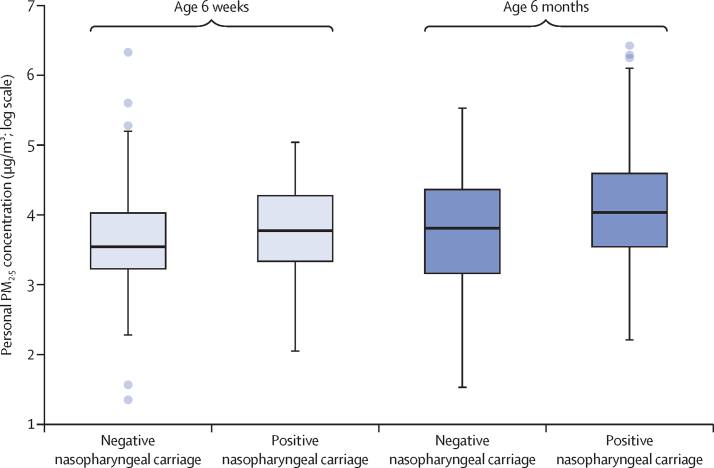
Box plot showing the distributions of average PM_2·5_ exposure by nasopharyngeal *Streptococcus pneumoniae* carriage status in children aged 6 weeks and 6 months Results are reported on a log scale. The thick black lines inside the boxes indicate the median values, the box representes the IQR, the whisker limits span 1·5 × the IQR, and the filled circles are outliers. PM=particulate matter.

The association between exposure to PM_2·5_ and nasopharyngeal *S pneumoniae* carriage was examined in 443 children (645 datapoints) with available information on exposure data, *S pneumoniae* carriage, household air pollution, vaccine status, and demographics. The distribution of PM_2·5_ exposure in children with and without *S pneumoniae*, stratified by age group, is shown in figure 2. At 6 weeks of age, geometric mean PM_2·5_ exposure in *S pneumoniae*-positive children (42·2 μg/m^3^ [95% CI 36·6–48·6]) was non-significantly higher than in *S pneumoniae*-negative children (38·2 μg/m^3^ [33·5–43·6]; p=0·13). At the age of 6 months, a significant difference in geometric mean PM_2·5_ exposure between *S pneumoniae-*positive children (60·3 μg/m^3^ [55·8–65·3]) and *S pneumoniae*-negative children (47·0 μg/m^3^ [38·3–57·7]; p=0·044) was observed).

The median duration of MicroPEM run-time was 47·91 h (IQR 8·14) in the control group and 47·98 h (6·81) in the intervention group.

The exposure-response analysis excluded 24 missing measurements. A significant exposure-response association between exposure to PM_2·5_ (categorised into deciles to maximise the efficiency of the analysis) and the prevalence of nasopharyngeal *S pneumoniae* carriage was observed in all study children (table 4). A one unit increase in decile of PM_2·5_ was found to significantly increase the risk of *S pneumoniae* carriage by 10% (OR 1·10 [95% CI 1·01–1·20], p=0·035), after adjustment for age, sex, PCV13 vaccination status, season, current use of antibiotics, and duration of MicroPEM run-time. In the intention-to-treat analysis, none of these confounders, other than male sex (0·41 [0·22–0·77]), were significantly associated with *S pneumoniae* carriage. When stratified by age group, a similar, albeit non-significant, exposure-response association was observed in children aged 6 weeks (1·06 [0·96–1·18]; p=0·24), and a significant association was observed in children aged 6 months (1·12 [1·01–1·25]; p=0·040). When the adjusted analysis was restricted to children with an exposure duration of 44–52 h (using the same covariates other than MicroPEM run-time), there was little change in the effect estimate (1·10 [0·99–1·22], p=0·069).

**Table 4:** Exposure-response association between exposure to PM_2·5_ and prevalence of nasopharyngeal *Streptococcus pneumoniae* carriage

	**Children aged 6 weeks (n=216)**		Children aged 6 months*(n=429)		All children*(n=645)	
	OR (95% CI)	p value	OR (95% CI)	p value	OR (95% CI)	p value
Crude estimates	1·07 (0·97–1·19)	0·18	1·10 (0·98–1·23)	0·093	1·16 (1·08–1·24)	<0·0001
Adjusted estimates†	1·06 (0·96–1·18)	0·24	1·12 (1·01–1·25)	0·042	1·10 (1·01–1·20)	0·035

OR=odds ratio. PM=particulate matter.

* Analysis accounts for non-independence of repeated measurements for children recruited at both 6 weeks and 6 months.

† Adjusted for age, sex, vaccination status, season, current antibiotic use, and duration of MicroPEM run-time.

*S pneumoniae* density data were excluded for 37 *S pneumoniae*-positive samples (involving 12 children at the age of 6 weeks and 25 children at the age of 6 months) because the results were invalid. The distribution of *S pneumoniae* density in study children was highly positively skewed, with a mean of 83·5 CFU/mL (SD 407·4). Average *S pneumoniae* density was higher in children aged 6 months (mean 93·7 CFU/mL [95% CI 47·9–139·5]) than in children aged 6 weeks (36·2 CFU/mL [8·5–63·8]), although this difference was not significant (p=0·26). *S pneumoniae* density was not associated with exposure to household air pollution in the study children (table 5). Analyses of exposure to PM_2·5_ (summarised in deciles) at 6 months of age (coefficient 1·04 [0·94–1·15], p=0·45), and in children aged 6 weeks and 6 months combined (0·99 [0·90–1·08], p=0·80), found no evidence of an exposure-response association. The small number of samples available for children recruited at the age of 6 weeks (n=79) precluded a robust exposure-response model in this age group.

**Table 5:** Association between *Streptococcus pneumoniae* density and exposure to PM_2·5_

	**All datapoints(n=427)**		**Children aged 6 months (n=348)**	
	Coefficient (95% CI)	p value	Coefficient (95% CI)	p value
Crude estimates	0·98 (0·89–1·08)	0·64	1·02 (0·92–1·12)	0·74
Adjusted estimates*	0·99 (0·90–1·08)	0·80	1·04 (0·94–1·15)	0·45

Data were generated with a multilevel mixed-effects linear regression model, with PM_2·5_ summarised in deciles. PM=particulate matter.

* Adjusted for age, sex, number of any vaccine doses received, season, and MicroPEM run-time.

## Discussion

To our knowledge, this is the first study to examine the effect of exposure to household air pollution, measured as PM_2·5_, on nasopharyngeal carriage of *S pneumoniae* in young children aged up to 6 months. The finding of an exposure-response association between exposure to PM_2·5_ and risk of *S pneumoniae* highlights a possible mechanistic pathway for the well documented association between household air pollution and ALRI in children.

In this sample of Malawian children, we found that over 85% had nasopharyngeal colonisation with *S pneumoniae* by the time they had reached 6 months of age. Compared with our study, other studies of children in Malawi have reported lower prevalence estimates of *S pneumoniae* at 6 months, including in both urban (50%)^23^ and rural (55%)^24^ contexts. A systematic review of studies done in sub-Saharan African countries, however, reported large variations in the prevalence of *S pneumoniae* carriage in this region*,* ranging from 21% in The Gambia to 93% in Ethiopia.^11^ Another study in Kenya found that the prevalence of *S pneumoniae* carriage peaked at 80% in children by the age of 6–11 months, with the prevalence gradually declining with age after this point.^25^ Our findings for *S pneumoniae* prevalence in children aged 6 weeks and 6 months are consistent with the range of values reported in sub-Saharan Africa.

The MSCAPE study took advantage of an ongoing randomised controlled trial in Malawi (CAPS), which was designed to investigate whether a cleaner-burning biomass-fuelled cookstove would reduce the incidence of pneumonia in young children.^18^ By recruiting children from within the CAPS structure and objectively measuring both nasopharyngeal carriage of *S pneumoniae* and exposure to PM_2·5_ in the recruited children, MSCAPE provided the opportunity for unique robust analysis of this association. This analysis included summarising the association between household air pollution exposure and *S pneumoniae* carriage both through an intention-to-treat analysis (in relation to the improved combustion cookstove intervention) and an exposure-response analysis. CAPS was the largest randomised controlled trial investigating the effect of an improved cookstove intervention on the incidence of pneumonia in young children, and the exposure data collected for the MSCAPE study represents one of the largest datasets of directly measured personal exposure to PM_2·5_ in children to date. PM_2·5_ is the single most important health-damaging constituent of household air pollution,^6^ and direct measurement is crucial to the study of associations between exposure and respiratory health outcomes, including the potential role of *S pneumoniae* carriage in ALRI development in children.

Our finding of no difference in the prevalence of *S pneumoniae* carriage between the intervention and control groups in the intention-to-treat analysis is consistent with the findings of CAPS.^18^ The probable explanation for the absence of an association in both studies could be that no difference in exposure to household air pollution was observed between the intervention and control groups, which has been previously reported for carbon monoxide,^26^ and now also for PM_2·5_.

Our exposure-response analysis found a significant association between personal exposure to PM_2·5_ and *S pneumoniae* colonisation, with an increase in decile of exposure to PM_2·5_ (natural log) associated with a 10% increased risk of *S pneumoniae* carriage (OR 1·10 [95% CI 1·01–1·20]), after adjusting for key confounders. However, we did not find an association between exposure to PM_2·5_ and of *S pneumoniae* density.

Several studies have examined the association between air pollution and nasopharyngeal carriage of respiratory pathogens, including *S pneumoniae*, in both adults (typically mothers) and children. These studies fall into three groups that can be defined by the following exposure sources: (1) household air pollution; (2) active and passive smoking; and (3) ambient (outdoor) air pollution. The studies of household air pollution and smoking have most in common, because the exposure is to biomass combustion in both studies. Ambient air pollution consists of pollutants from a wider range of sources, including vehicles (diesel and petrol), industry, power generation, and biomass. PM_2·5_ is regarded as the key health-damaging pollutant from all of these sources, household air pollution and tobacco smoking included.

We are aware of just two other studies, both from sub-Saharan Africa, reporting on the association between exposure to household air pollution and nasopharyngeal pneumococcal carriage. Litchfield^16^ did a randomised control trial in The Gambia comparing the effect of using alternative stoves with biomass briquettes as fuel with standard wood-burning stoves with wood as fuel on nasopharyngeal carriage of pneumococcus among 252 pairs of children aged younger than 5 years and their mothers. The prevalence of pneumococcus carriage was 24·9% in mothers and 74·6% in children. Mean exposure to PM_2·5_ during a 48-h period was almost twice as high in the kitchens of pneumococcal-positive versus pneumococcal-negative children (p<0·001). Analysis by quintile of PM_2·5_ showed a higher prevalence of nasopharyngeal pneumococcal carriage in children with higher pollution exposure concentrations (p<0·01). However, this association was tested only for independence across quintiles and not for trend, and was not adjusted for confounding.

Carrión and colleagues^15^ report the results of the randomised control GRAPHS trial done in rural Ghana, in which the association between household air pollution and nasopharyngeal carriage of *S pneumoniae* in children was investigated. The trial was designed to compare the effect of using a clean cooking fuel (liquefied petroleum gas) and an improved cookstove (a Biolite fan stove; intervention group) with using wood on a three-stone fire (control group) on the incidence of ALRI in children. The analysis of *S pneumoniae* carriage was done in a subgroup of 130 children with pneumonia and 130 healthy controls to compare nasopharyngeal carriage of 22 bacterial and viral pathogens between the two groups.^15^ Exposure assessment relied on carbon monoxide because, although PM_2·5_ was measured in some trial participants, too few of the 260 children selected for the *S pneumoniae* analysis had these data. In the intention-to-treat analysis, the children from households in the control group had a significantly higher nasopharyngeal microbial count than those in the intervention group (p<0·0001), a finding driven by bacterial rather than viral pathogens. Comparison of *S pneumoniae* carriage in children from control group households versus those from intervention group households reported an OR of 2·42 (95% CI 1·07–5·81; p value not reported). The intention-to-treat analysis of the most recent postnatal carbon monoxide exposure value, however, found that this value was higher in intervention group households than in control group households. The opposite association was found for prenatal carbon monoxide measurements (no p values for either association were provided). Adjusted analysis of the association between carbon monoxide exposure and bacterial carriage found no significant results. The correlation between carbon monoxide and PM_2·5_ exposure in the GRAPHS trial sample was quite weak (Spearman's *r*=0·212), but was significant (p=0·016). The authors concluded that using carbon monoxide as a measure of exposure to household pollution might not capture all of the pollutants that have any real effects on nasopharyngeal pneumococcal carriage. The apparent inconsistency between the intention-to-treat findings for postnatal exposure differences (ie, the higher carbon monoxide exposure in the intervention group *vs* the control group) and bacterial carriage (ie, the higher prevalence in the control group *vs* the intervention group) was not discussed further.^15^

More extensive studies are available on the association between *S pneumoniae* carriage and both active and passive (environmental) tobacco smoking.^13^, ^14^, ^27^, ^28^, ^29^ Some of these studies also report findings for measured household air pollution or fuel used for cooking. One of the largest of these studies was done in South Africa and enrolled almost 1000 pregnant women.^14^ A third of the mothers smoked, and 44% of mothers and 69% of their children were exposed to environmental tobacco smoke. Household air pollution was assessed with measurements including carbon monoxide and PM_10_ concentrations. None of the 911 infants had nasopharyngeal carriage of *S pneumoniae* at birth, 584 (66%) of 887 had carriage at age 6 months, and 547 (68%) of 800 at age 12 months. Despite a low overall prevalence of *S pneumoniae* carriage in mothers (6%), active smoking was found to significantly increase the risk of *S pneumoniae* carriage, with an adjusted risk ratio of 1·73 (95% CI 1·03–2·92; adjusted for weight-for-age Z score at birth, preterm birth, ethnicity, sex, HIV exposure, time on exclusive breastfeeding, average number of people per sleeping room, dwelling category, recent respiratory infection, day care attendance, vaccination status, number of other children under 5 years in the household, and antibiotic use). Environmental tobacco smoke exposure increased the risk of *S pneumoniae* carriage in children aged 6 months (1·14 [1·00–1·30]), as did exposure to household air pollution, measured by carbon monoxide concentrations (that were higher than an ambient standard threshold; 1·33 [1·03–1·72]; risk ratios were adjusted for the same aforementioned factors and exposure to other pollutants). These findings for smoking are reflected in several other studies, although the strength and consistency of findings vary between studies. A systematic review and meta-analysis examining risks of smoking and pneumococcal carriage would help to interpret the findings of these studies, some of which might not be sufficiently powered.

A study of 208 children aged 5 years or younger and their mothers in southern Israel, which was done before introduction of the pneumococcal vaccine, reported a significantly higher prevalence of *S pneumoniae* carriage in children exposed to environmental tobacco smoke than those who were not exposed, and in mothers who smoked or were exposed to environmental tobacco smoke compared with those who did not smoke or were not exposed.^27^

In Ethiopia, a study of risk factors for *S pneumoniae* carriage among 362 children with community-acquired pneumonia aged 15 years or younger (median age 9 months) reported findings for exposure to environmental tobacco smoking and cooking fuel.^30^ The prevalence of parental smoking was low (3·3%), but the results of the adjusted analysis found an OR for *S pneumoniae* carriage in children exposed to tobacco smoke versus those who were not exposed of 3·0 (95% CI 0·90–9·97; p=0·073). The results for exposure to polluting cooking fuels (wood and charcoal) versus clean cooking fuels (gas and electricity), in a new analysis done by us, produced an OR of 1·14 (0·73–1·79; p=0·53). In one study done in Cyprus, Koliou and colleagues^28^ reported a non-significant association between *S pneumoniae* carriage and exposure to environmental tobacco smoke in 402 children aged 6 months to 5 years (adjusted OR 1·43 [0·86–2·40]; OR adjusted for gender, age, parent nationality, previous or current breastfeeding, number of siblings, attendance at day care centres, immunisation with PCV7 vaccine, specimen origin, and recent use of antibiotics), of whom 142 (35·3%) children had been exposed.

Finally, a study of 1125 children aged 2–6 years from northeastern Iran, with a low prevalence of *S pneumoniae* carriage (10·1%) and with only 165 (14·7%) of 1125 exposed to environmental tobacco smoking, reported a non-significant adjusted OR of 1·08 (0·60–1·22; p=0·80; OR adjusted for age, sex, allergic rhinitis, number of siblings, breastfeeding, and economic status of the family).^29^

To our knowledge, there is only one study published to date investigating the association between exposure to ambient air pollution and nasopharyngeal carriage of *S pneumoniae* in children aged younger than 6 months, known as the Generation R Study.^13^ The authors found a significant increase (of 10%) in the prevalence of *S pneumoniae* with each one unit (10 μg/m^3^) increase in exposure to PM_10_ compared with the previous day (OR 1·12 [95% CI 1·00–1·26]; p<0·05).

There are some limitations of the MSCAPE study that need to be considered. At the time that this study was done, it was not known that the intervention cookstove adopted in the main CAPS was not leading to significant reductions in exposure to household pollution when compared with controls. Indeed, compliance in the intervention group was found to be low after the study was completed. This low compliance affected the intention-to-treat analysis of nasopharyngeal *S pneumoniae* carriage in the MSCAPE study due to the absence of an intervention effect. However, the exposure-response analysis in the MSCAPE study was done independently from the CAPS intervention. Another limitation concerned the difficulty in measuring personal exposure to PM_2·5_ (the most important variable for the MSCAPE study) in young children. The record of everyday exposure in the MSCAPE study children will include a degree of random error due to high intra-child variation in exposure over time. In addition, the sampling duration using the air pollution monitoring equipment (MicroPEM) varied between the study children. Ideally, the sampling period should have been 24 h or 48 h for each child, representing a full day or 2 days of cooking in the household, but in practice, recording periods were both shorter and longer than these two discrete periods. To account for the effect of differences in sampling duration on the study results, we adjusted for sampling duration in a sensitivity analysis, in case sampling over longer periods spuriously resulted in lower or higher average levels of exposure than expected. We found no evidence that sampling duration affected the amount of exposure or the exposure-response analysis. Another limitation of the MSCAPE study concerned the sample size. Additional children were recruited at 6 months, who were not seen at 6 weeks, to ensure the required sample size was achieved before the intervention was given to the control households. The sample of children aged 6 weeks remained underpowered for analysis.

In conclusion, we found that exposure to higher concentrations of PM_2·5_, the single most important health-damaging pollutant in household air pollution, is associated with increased prevalence of nasopharyngeal *S pneumoniae* colonisation in young children. Household air pollution is a known risk factor for childhood pneumonia, and nasopharyngeal colonisation is a necessary step in the progression to invasive pneumococcal disease. This finding, which is consistent with other studies of exposure to cooking-derived smoke pollution, active and passive smoking, and ambient air pollution, provides some important new evidence of intermediary steps in the causal pathway of household air pollution exposure to pneumonia. Further studies, particularly new randomised controlled trials comparing clean fuels (eg, liquefied petroleum gas) with biomass fuels, with detailed measurements of PM_2·5_ exposure, and studies of mechanisms underlying increased pneumococcal carriage, are required to strengthen causal evidence for this component of the pathway from household air pollution exposure to ALRI in children.

## Data sharing

The study data are deposited at the University of Liverpool repository. All anonymised data, along with the data dictionary, study protocol, and consent forms, will be available from the corresponding author immediately after publication. The data will be available to anyone who wishes to carry out further research and shares the protocol and methods of their study. After a request is made, the study governing committee (MKD, NGB, DP, and NF) will decide whether to grant access to the data on a case-by-case basis, and will share data after signing a data access agreement with the requesting body.

## Declaration of interests

We declare no competing interests.
